# Healing of intrabony defects following regenerative surgery by means of single-flap approach in conjunction with either hyaluronic acid or an enamel matrix derivative: a 24-month randomized controlled clinical trial

**DOI:** 10.1007/s00784-021-03822-x

**Published:** 2021-02-10

**Authors:** Andrea Pilloni, Mariana A. Rojas, Lorenzo Marini, Paola Russo, Yoshinori Shirakata, Anton Sculean, Roberta Iacono

**Affiliations:** 1 Section of Periodontology, Department of Oral and Maxillofacial Sciences, Sapienza University of Rome, 6 Caserta Street, 00161 Rome, Italy; 2 Department of Periodontology, Kagoshima University Graduate School of Medical and Dental Sciences, Kagoshima, Japan; 3 Department of Periodontology, School of Dental Medicine, University of Bern, Bern, Switzerland

**Keywords:** Enamel matrix derivative, Intrabony defects, Hyaluronic acid, Periodontal pocket, Periodontal regeneration, Randomized clinical trial

## Abstract

**Objectives:**

The aim of this randomized controlled clinical trial was to compare the clinical outcomes obtained in intrabony defects following regenerative periodontal surgery using the single-flap approach (SFA) in conjunction with either hyaluronic acid (HA) or enamel matrix derivative (EMD).

**Materials and methods:**

Thirty-two intrabony defects in 32 healthy subjects were randomly assigned: HA (test group) or EMD (control group). Clinical attachment level (CAL), probing depth (PD), gingival recession (REC), and bleeding on probing (BOP) were recorded at baseline,12, 18, and 24 months after surgery.

**Results:**

At 24 months, both treatments resulted in statistically significant clinical improvements evidenced by PD-reduction and CAL-gain (*p*<0.001). The mean CAL-gain was 2.19±1.11 mm in the test and 2.94±1.12 mm in the control sites (*p*=0.067). PD-reduction was statistically significantly higher for the control group (4.5±0.97 mm) than the test group (3.31±0.70 mm), (*p*=0.001). CAL-gain ≤ 3 mm was observed in 87.5% and in 62.5% of the test and control sites, respectively. Test sites showed slightly lower REC values than the control sites. No statistically significant differences were found for BOP between treatments.

**Conclusions:**

The present findings indicate that both treatments led to statistically significant clinical improvements compared to baseline, although the application of EMD resulted in statistically significantly higher PD-reduction compared to the use of HA.

**Clinical relevance:**

The use of HA in conjunction with a SFA resulted in significant PD-reduction and CAL-gain, pointing to the potential clinical relevance of this material in regenerative periodontal surgery.

## Introduction

Periodontitis may lead to the formation of intrabony defects, defined as specific osseous defects with a base apical to the interdental alveolar crest and surrounded by one, two, or three bony walls or a combination thereof [1]. If left untreated, these defects represent a risk factor for disease progression and additional attachment and bone loss [2]. Surgical intervention is considered the treatment of choice for deep intrabony defects, which have not resolved following completion of cause-related periodontal therapy [3]. Regenerative procedures including the use of certain types of bone replacement materials, barrier membranes, enamel matrix derivative (EMD), recombinant platelet-derived growth factor (rhPDGF), or various combinations thereof have been shown to facilitate periodontal regeneration characterized histologically by formation of root cementum, periodontal ligament, and alveolar bone and to result superior in clinical, radiographical, and patient-reported outcomes compared to access flap surgery alone [4, 5]. Furthermore, the use of specific flap designs with maximum preservation of the interdental soft tissue or limiting flap elevation by means of a single-flap approach (SFA) [6], when the location and configuration of the defect advise it, is highly recommended to optimize wound stability and reduce morbidity [5, 6].

Hyaluronic acid (HA), an anionic, non-sulfated glycosaminoglycan structured biomolecule, is a major component of the extracellular matrix and can be found in almost all organs and tissues including the periodontium. In the periodontal tissues, it is synthetized by fibroblasts and keratinocytes in the gingiva and by periodontal ligament cells, cementoblasts, and osteoblasts [7, 8].

HA is known for being extremely hygroscopic and viscoelastic and for its essential role in maintaining the structural and homeostatic integrity of tissues. It was demonstrated that HA has bacteriostatic [9, 10], fungostatic [11], anti-inflammatory [12], anti-edematous [13], osteoinductive [12, 14–16], and pro-angiogenetic [17] properties. Furthermore, it plays a significant role in inflammation, clot and granulation tissue formation, cell migration and differentiation during tissue formation, and repair of both soft and hard tissues [18, 19].

Data from a recent in vitro study have provided evidence on the effects of HA to maintain the viability of oral fibroblasts and increase of their proliferative and migratory abilities. Furthermore, HA enhanced the expression of genes encoding type III collagen and transforming growth factor-β3, characteristic of scarless wound healing, and upregulated also the expression of genes encoding pro-proliferative, pro-migratory, and pro-inflammatory factors [20]. Very recently, it has been also shown that HA strongly induces the growth of osteoprogenitors and maintains their stemness, thus suggesting a potential regulatory effect of HA on the balance between self-renewal and differentiation during bone healing/regeneration [21]. When used as a physical background material, HA functions also as space filler, lubricant, and a protein excluder as well [22].

The potential effects of HA on periodontal wound healing/regeneration has been very recently evaluated histologically in experimentally created two-wall intrabony defects in dogs [23]. The study has for the first time provided histological evidence for the formation of root cementum, periodontal ligament, and bone following the application of HA in conjunction with periodontal surgery, thus suggesting that the clinical improvements reported following the use of this material may indeed reflect periodontal regeneration [23].

Taken together, the available data suggest that HA may represent a promising material for periodontal regenerative/reconstructive surgery. Up to date, two systematic reviews (i.e., only one with a meta-analysis) have been published on the effects of HA when used in conjunction with surgical periodontal therapy. The results have shown that HA in conjunction with access flap may provide positive effects demonstrated by an additional reduction in probing depth (PD) and clinical attachment level (CAL) gain in intrabony defects compared with access flap alone [24, 25].

One of the most widely and best documented regenerative materials is an enamel matrix derivative (EMD). When used in conjunction with regenerative periodontal surgery, EMD has been shown to promote periodontal regeneration and lead to substantial clinical improvements evidenced by gain of clinical attachment level (CAL), probing depth (PD) reduction, and eventually hard tissue fill, thus improving long-term tooth prognosis [26–28]. Today, EMD is generally considered one of the standard regenerative materials recommended for the treatment of intrabony defects [29].

According to the best of our knowledge, until now, no clinical studies have evaluated the effects of HA when used in conjunction with reconstructive periodontal surgery in intrabony defects, and compared those to the outcomes obtained following the use of EMD.

Therefore, the aim of this randomized controlled clinical trial was to compare the clinical outcomes obtained in intrabony defects following regenerative periodontal surgery using the single-flap approach (SFA) [6] in conjunction with either HA or EMD.

## Materials and methods

### Study design

A randomized controlled clinical trial was conducted to test the efficacy of hyaluronic acid used in conjunction with regenerative surgery in intrabony defects by means of the SFA. The test group included the application of cross-linked HA—HA gel composed of a mixture of cross-linked (1.6%) and natural (0.2%) hyaluronic acid (hyaluronic acid, hyaDENT BG, Bioscience, Germany)—while the control group was treated with EMD (Straumann Emdogain, Straumann, Basel, Switzerland). The same surgical procedures were performed in both groups. The clinical outcomes were assessed at baseline and 12, 18, and 24 months post-surgery.

**Fig. 1:**
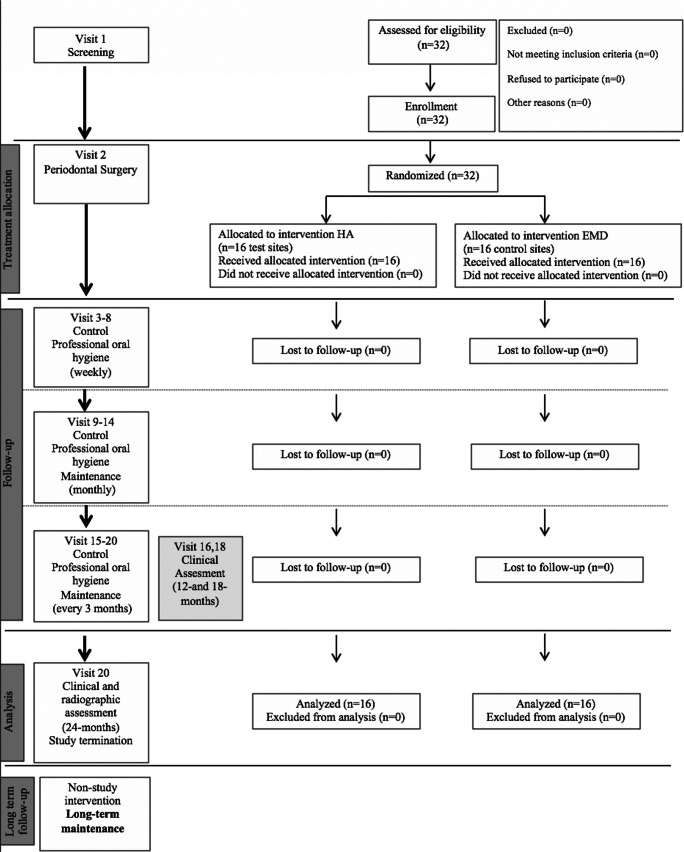
CONSORT flow chart for patient enrollment, allocation, follow-up, and analysis

The study was performed according to current standards of clinical research (CONSORT guidelines) (http://www.consort-statement.org). The CONSORT diagram is presented in Fig. 1.

The study protocol (ClinicalTrial.gov- NCT04319770) was approved by Ethical Committee of “Sapienza,” University of Rome (Ref. 3816 – Prot. 2705/15).

The protocol was performed in accordance to the Good Clinical Practice (GCPs) guidelines (1996) and Declaration of Helsinki of 1975, as revised in 2013.

### Study population

From September 2016 until October 2019, all patients undergoing periodontal treatment at the clinical centre in the Section of Periodontology, Sapienza University of Rome, Department of Oral and Maxillofacial Sciences, were screened for this study.

The following inclusion criteria were applied: (1) adults aged 18–65 years with periodontal disease, (2) good physical health, (3) sites with intrabony defects on single-rooted teeth and persisting pockets (probing depth (PD) ≥ 6 mm and bleeding on probing (BOP)) at re-evaluation 6 weeks after non-surgical periodontal therapy, (4) radiographic intrabony component ≥ 3 mm, (5) limited to no extension of the defect on the lingual or palatal side as assessed by preoperative bone sounding [5], and (6) full-mouth plaque score (FMPS) and full-mouth bleeding score (FMBS) ≤ 20% before surgery [30, 31].

Patients were excluded on the basis of the following: (1) relevant medical conditions contraindicating surgical interventions, (2) pregnancy or lactation, (3) tobacco smoking, (4) untreated periodontal conditions, (5) any condition associated with poor compliance or failure to maintain good oral hygiene, (6) acute infectious lesions in areas intended for surgery, (7) teeth with grade 2 or higher mobility, (8) restorations or carious lesions on root surfaces that are associated with the intrabony defect.

All patients signed a written informed consent before participation.

### Sample size

The study was designed for superiority and the sample size was calculated assuming *α* = 0.05 and the power of sample (1−*β*) = 90%.

Considering the absence of previous studies providing data on beneficial effect of hyaluronan gel on the CAL, the sample size was calculated based on data from a study on the effect of Emdogain on the CAL [26] assuming a difference of 0.5 mm between the means and a standard deviation of the difference in means of 0.35 mm.

Considering possible dropouts, the number of the patients was also increased by of 15% for each group. On the basis of the data and these assumptions, 16 patients for the test group and 16 for the control group were required to be entered in this study.

### Randomization and allocation concealment

Each patient was randomly assigned to one of the two groups. Allocation concealment was performed using opaque and sealed envelopes, which were sequentially numbered. The allocation sequence was determined using a computer-generated randomization list (IBM SPSS, Version 22.0, Chicago, IL, USA). One examiner (LM), who was not involved in the treatment sequence, was assigned to open the envelope, immediately after flap elevation. The treatment was communicated to the operator (AP).

### Experimental procedures

#### Clinical measurements

The following clinical measurements were taken at baseline and at 12, 18, and 24 months after surgery for each tooth by a blinded calibrated examiner (PR): (1) full-mouth plaque score (FMPS) [30] and (2) full-mouth bleeding score (FMBS) [31], recorded as the percentage of total surfaces (four aspects per tooth); (3) bleeding on probing (BOP) recorded dichotomously at surgical site as the presence or absence of bleeding; (4) probing depth (PD) and (5) gingival recession (REC), recorded to the nearest millimetre at the deepest location of the selected interproximal site; and (6) clinical attachment level (CAL) calculated as the sum of PD and REC.

Only pre-surgical and post-surgical measurements at the deepest site of the intrabony defect were considered for the future post-surgical analysis.

All the measurements were taken by using a calibrated periodontal probe (PCP-UNC 15, Hu-Friedy, Chicago, Illinois, USA).

#### Examiner calibration

Before the study was conducted, intra-examiner calibration was performed. Clinical measurements were obtained at two different time points separated by a 2-week interval. Intra-class correlation coefficients (ICC) was calculated, and the process was repeated until a good correlation was obtained (ICC ≥ 0.75).

#### Pre-surgical phase

Prior to surgery, all subjects were instructed to proper oral hygiene procedures and quadrant wise/full-mouth scaling and root planning with ultrasonic and manual instruments was performed. The patients were re-evaluated 6 weeks after completion of the initial therapy to determine their response to therapy and to confirm the need for periodontal surgery [32].

#### Surgical phase

**Fig. 2:**
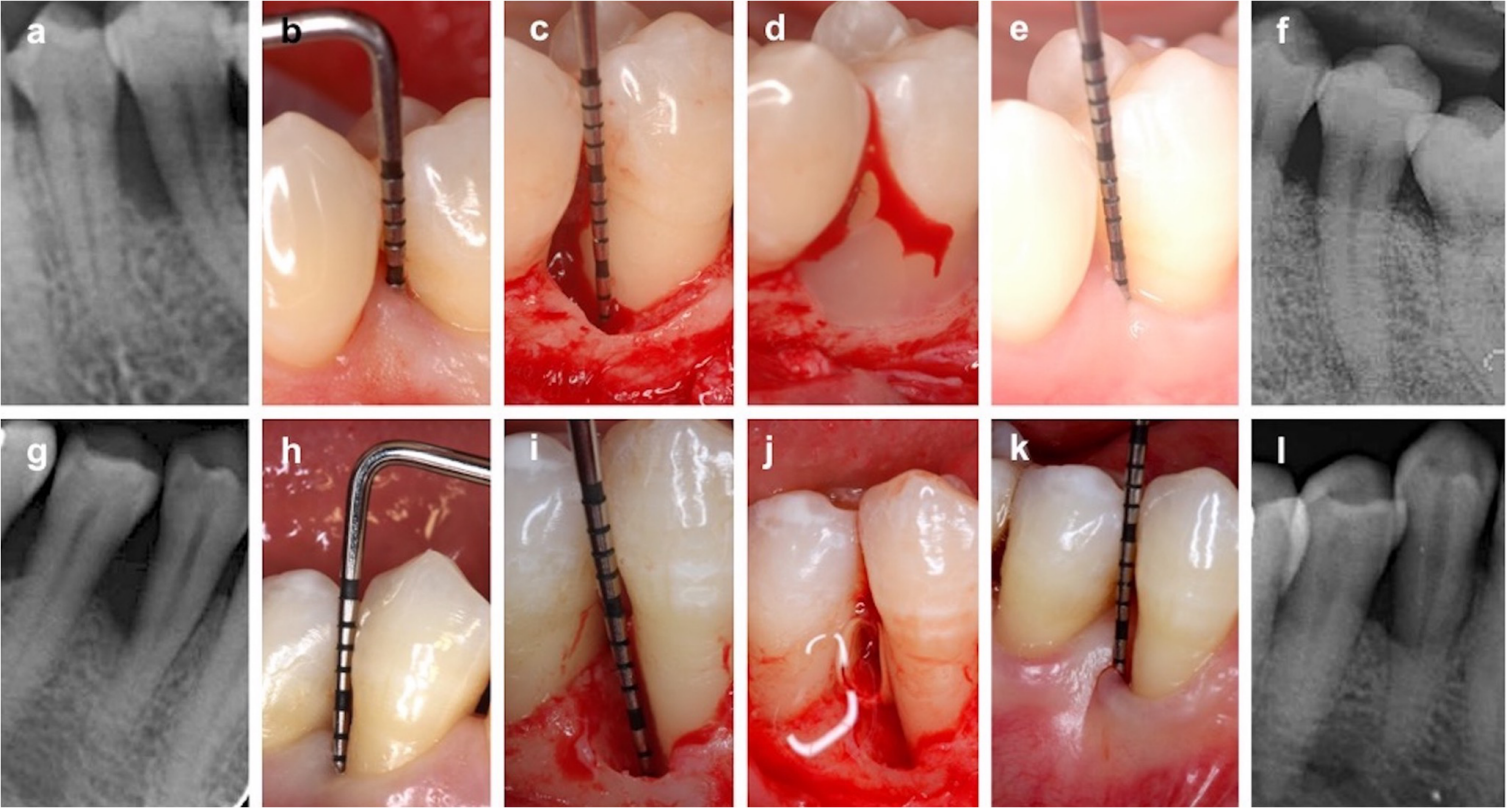
Illustration of representative control (**a**–**f**) and test (**g**–**l**) group cases. Control group case (EMD): **a** baseline radiographic view, **b** baseline clinical view. Intrabony defect on the mesial aspect of the mandibular left second premolar. **c** Intraoperative view of the defect, **d** Enamel matrix derivative (EMD) application. **e** 24-month follow-up clinical view. **f** 24-months follow-up radiographic view. Test group case (HA): **g** baseline radiographic view, **h** baseline clinical view. Intrabony defect on the distal aspect of mandibular right first premolar. **i** Intraoperative view of the defect, **j** Hyaluronic acid application, **k** 24-months follow-up clinical view. **l** 24-months follow-up radiographic view

Representative control and test group cases are shown in Fig. 2.

All surgical procedures were performed by the same experienced operator (AP) with more than 10 years of experience.

After local anesthesia, bone sounding was performed pre-surgically to determine the characteristics of the bony defect, including the defect morphology and extension. Surgical access was obtained using the SFA, which involves the elevation of a flap on only one aspect of the defect (i.e., either buccal or oral, depending on the extension/morphology of the lesion), thus preserving the integrity of the interdental soft tissue. Briefly, the buccal gingival tissue was incised at least one tooth mesial and distal to the defect site to provide access for visualization and instrumentation of the defect. Vertical releasing incisions were placed mesial or distal to the treated defect, if they were considered necessary to improve visibility and/or to achieve a tension free flap closure.

After flap reflection, granulation tissue was removed from the defect and it was examined to confirm its anatomy, as outlined by the preoperative radiograph. Scaling and root planing were performed with hand and ultrasonic instruments and the defect was rinsed with sterile saline solution.

After site preparation, the sealed envelope was opened, and the defect was assigned to the HA (test group) or EMD (control group) treatment. The root surfaces of teeth from control group were conditioned for 2 min with EDTA gel (sterile 24% EDTA gel, pH 6.7; PrefGel, Straumann, Basel, Switzerland) in order to remove the smear layer [33]. Then, to remove any EDTA residue, the defect and the adjacent soft tissues were thoroughly rinsed with sterile saline solution. The EMD gel was applied on the root surfaces and into the intrabony defect. In the test group, HA was applied directly into the defect by means of a sterile syringe.

In both treatment groups, the periosteum at the base of the flaps was gently dissected to allow tension release.

Finally, flaps were positioned at the pre-surgical level or slightly coronal without any tension. Monofilament non-resorbable 6-0 nylon suturing material was used (Ethilon‚ Ethicon, Johnson & Johnson, Somerville, NJ, USA). Selection of the suturing technique was on the basis of the flap design, according to the indications proposed by the authors [6, 34], i.e., a horizontal internal mattress suture at the base of the papilla and a second internal mattress suture (vertical or horizontal) between the most coronal portion of the flap and the most coronal portion of the palatal/lingual papilla. In case of large, thick interdental papilla, an interrupted suture was performed to ensure primary intention healing.

Extreme care was taken to obtain primary closure of the interdental soft tissues.

#### Post-surgical instructions and plaque control

The patients received systemic antibiotic therapy (Amoxicillin) 2 g/day for 6 days. Pain control was obtained by 400 mg Ibuprofen 3 times per day for the first 72 h and subsequent doses were indicated only if needed.

All the patients were advice to rinse twice per day with a 0.12% chlorhexidine gluconate solution (Periodex, Zila Pharmaceuticals Inc., Phoenix, AZ) for 6 weeks and mechanical tooth cleaning was not allowed in the surgical area during this period.

Sutures were removed at 10–12 days following surgery and the patients were instructed to brush with a post-surgical soft toothbrush. The use of a soft toothbrush was discontinued only after the 3-month follow-up. Then, a medium-sized bristle toothbrush was prescribed.

#### Follow-up and re-evaluation

Patients were recalled weekly for the first 6 weeks to perform gentle supragingival professional tooth cleaning and reinforcement of oral hygiene. Afterwards, the patients were enrolled into monthly recall visits during the first 6 months, and every 3 months thereafter. At the follow-up visits, professional oral hygiene/maintenance procedures and oral hygiene instructions reinforcement were performed. Probing and subgingival instrumentation will not be performed in ≤12-month re-evaluation.

#### Outcome measures

The primary outcome measure defined in the study was the change in CAL between the test and control sites. Secondary outcome measures outlined were changes in PD, REC, and BOP.

### Statistical analysis

All data analysis was carried out according to a pre-established analysis plan by a biostatistician blinded to group allocation.

The descriptive and statistical analyses were performed using a validated software (R software, version 3.6.1.) Data were expressed as mean ± standard deviation, median, and interquartile range and were calculated by treatment (HA, test group and EMD, control group) and time (baseline, 12-, 18- and 24-months). The normal distribution of data in each observation period was assessed through the Shapiro-Wilk test (SW test). Bartlett’s test was used to test the homogeneity of variances.

The significance level was set at 0.95. If the hypothesis of normality was confirmed (*p* value*>* 0.05*)*, *t*-test was performed to detect and to analyze the difference between HA group and EMD group. If the hypothesis of normality was rejected, the Wilcoxon-Mann-Whitney test was used to compare the two groups.

Moreover, the analysis of the result for each treatment (HA or EMD) was performed with Wilcoxon’s test for paired sample.

Finally, repeated measures ANOVA was used to determine the treatment-time interactions within each group.

## Results

### Study population

Thirty-two systemically healthy adult subjects, 16 in each group, were included in the present study. The study population consisted of 17 females and 15 males, aged 28 to 60 years with mean age at baseline of 41.47 years ± 9.25. In the HA group, there were 8 females and 8 males and mean age was 41.19 ± 8.49; EMD group included 9 females and 7 males with a mean age of 41.75 ± 10.22. Demographic data are summarized in Table 1.

**Table 1:** Individual patient characteristics and baseline clinical parameters

Demographic data	HA (*n*=16)	EMD (*n*=16)	*p* value
Age	41.19 ± 8.49	41.75 ± 10.22	0.94^a^
Gender (female/male)	8/8	9/7	0.74^a^
Clinical parameter			
FMPS (%)	12.27 ± 2.66	11.41± 2.45	0.32^a^
FMBS (%)	12.81 ± 2.86	11.93 ± 3.45	0.44^a^
CAL (mm)	7.37 ± 0.88	7.37 ± 0.96	0.97^b^
PD (mm)	7.31 ± 0.97	7.25 ± 0.93	0.83^b^
REC (mm)	0.06 ± 0.68	0.12 ± 0.62	0.81^b^
BOP	0.19 ± 0.40	0.37 ± 0.5	0.24^a^

Performed statistical tests:

^a^Wilcoxon’s test

^b^Wilcoxon-Mann-Whitney test

All data are expressed as mean and standard deviation

*HA* hyaluronic acid, *EMD* enamel matrix derivatives, *FMPS* full-mouth plaque score, *FMBS* full-mouth bleeding score, *CAL* clinical attachment level, *PD* probing depth, *REC* recession depth, *BOP* bleeding on probing, *NS* not significant

Level of significance *p*<0.05

One intrabony defect was treated for each patient. In all cases, buccal SFA ensured adequate surgical access for root and defect instrumentation and primary intention wound healing was obtained. No relevant intraoperative or postoperative complications occurred in any of the patients.

All the patients completed the study, reaching the end of the follow-up period at 24 months.

### Baseline clinical parameters

Baseline clinical parameters are shown in Table 1. No statistically significant differences were observed among the baseline clinical parameters.

### Clinical parameters at 12, 18, and 24 months

Details of the clinical parameters are presented in Table 2. From baseline to 24 months, statistically significant improvements for CAL and PD were measured in both groups (*p*<0.001). REC was slightly increased in both groups while the BOP values reveal statistically significant changes neither within nor between the two groups.

- CAL. At 12, 18, and 24 months, the values decreased significantly in both groups. At 18 and 24 months, the comparison between the two groups showed significant differences (*p=* 0.047 and *p*= 0.0125, respectively).
- PD. At 12, 18, and 24 months, the values decreased significantly in both groups. At each time interval, the comparison between the two groups showed significant differences (*p*= 0.004 at 12 months; *p*= 0.002 at 18 months; and *p*< 0.001 at 24 months).
- REC. At 12, 18, and 24 months, the values increased significantly in both groups. The comparison between the two groups did not show significant differences.
- BOP. At 12, 18, and 24 months, the values did not significantly change in both groups.

**Table 2:** Clinical parameter values of test (HA) and control sites (EMD) at 12, 18, and 24 months

	12 months			18 months			24 months		
Parameter	Mean±SD	Median [IQR]	*p* value	Mean±SD	Median [IQR]	*p* value	Mean±SD	Median [IQR]	*p* value
CAL (mm)									
HA	4.94 ± 1.06	5.0 [1.0]	<0.001^a^*	5.19 ± 1.28	5.0 [2.0]	<0.001^a^*	5.19 ± 1.42	5.0 [2.0]	<0.001^a^*
EMD	4.25 ± 1.29	4.0 [2.0]	<0.001^a^*	4.31 ± 1.08	4.0 [1.0]	<0.001^a^*	4.44 ± 1.03	4.0 [1.0]	<0.001^a^*
HA vs EMD	-	-	0.085^b^	-	-	0.047^b^*	-	-	0.0125^a^*
PD (mm)									
HA	4.18 ± 0.81	4.0 [1.25]	<0.001^a^*	4.12 ± 1.14	4.0 [2.0]	<0.001^a^*	4.00 ± 1.09	4.0 [1.25]	<0.001^a^*
EMD	3.00 ± 1.22	3.0 [2.0]	<0.001^a^*	2.87 ± 0.80	3.0 [1.25]	<0.001^a^*	2.75 ± 0.57	3.0 [1.0]	<0.001^a^*
HA vs EMD	-	-	0.004^b^*	-	-	0.002^b^*	-	-	<0.001^a^*
REC (mm)									
HA	0.75 ± 0.58	1.0 [1.0]	<0.001^a^*	1.06 ± 0.57	1.0 [0.0]	<0.001^a^*	1.19 ± 0.75	1.0 [0.25]	0.002^a^*
EMD	1.25 ± 0.69	1.0 [0.25]	<0.001^a^*	1.44 ± 0.63	1.0 [1.0]	<0.001^a^*	1.69 ± 0.70	2.0 [1.0]	0.003^a^*
HA vs EMD	-	-	0.354^b^	-	-	0.110^b^	-	-	0.052^b^
BOP									
HA	0.25 ± 0.45	0	0.755^a^	0.19 ± 0.4	0	1^a^	0.19 ± 0.40	0	1^a^
EMD	0.31 ± 0.49	0	0.755^a^	0.37 ± 0.5	0	1^a^	0.31 ± 0.48	0	0.6547^a^
HA vs EMD	-	-	0.8^b^	-	-	0.87^b^	-	-	0.9^b^

Performed statistical tests:

^a^Wilcoxon’s test

^b^Wilcoxon-Mann-Whitney test

Data are expressed as mean and standard deviation and as medians and interquartile range values

*HA* hyaluronic acid, *EMD* enamel matrix derivatives, *SD* standard deviation, *IQR* interquartile range, *CAL* clinical attachment level, *PD* probing depth, *REC* recession depth, *BOP* bleeding on probing

*p value < 0.05 indicates statistically significant differences

### Clinical parameters changes at 12, 18, and 24 months

**Fig. 3:**
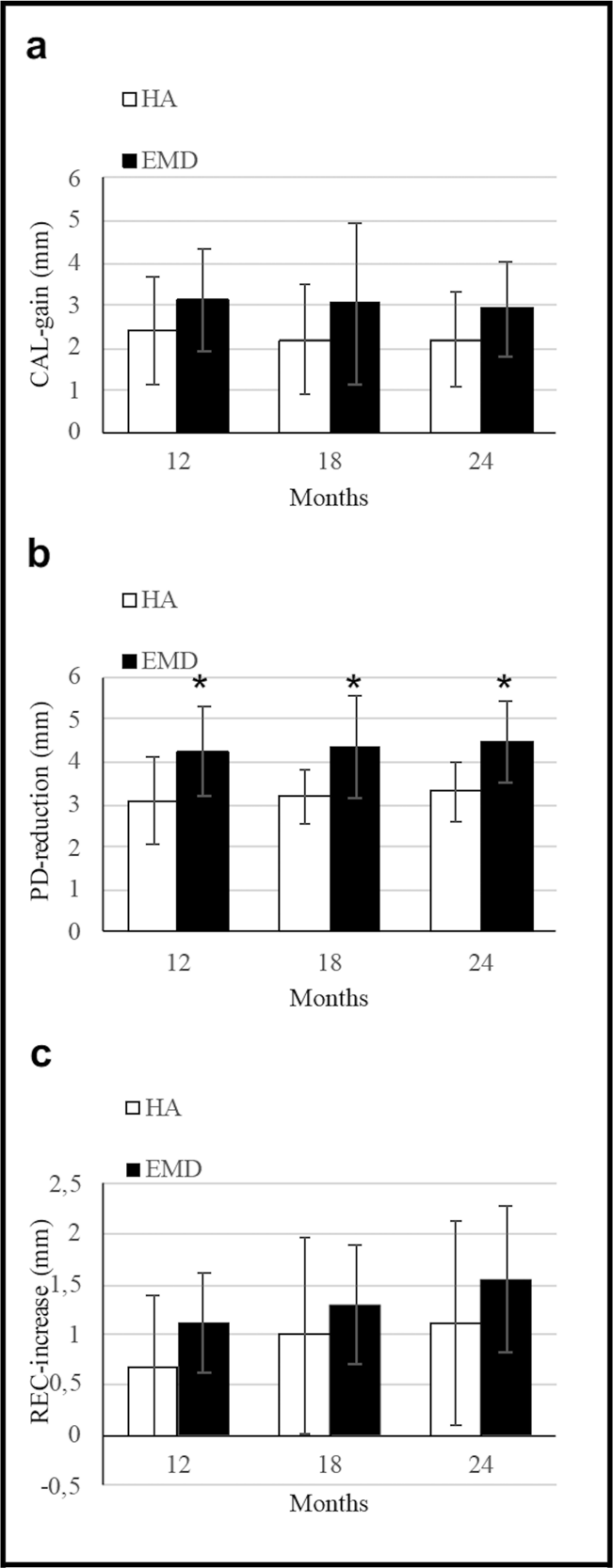
Mean values and standard deviations of clinical parameter changes in test (HA) and control sites (EMD) at 12, 18, and 24 months: **a** clinical attachment level gain (CAL-gain), **b** Probing depth reduction (PD-reduction), **c** recession increase (REC-increase). *Significant (*p*< 0.05) difference between test and control group

- CAL-gain. At 12, 18, and 24 months, the CAL-gain was slightly higher for the EMD group (3.12 mm ± 1.20 mm; 3.06 mm ± 1.29 mm; and 2.94 mm ±1.12 mm, respectively) than the HA group (2.43 mm ± 1.26 mm; 2.19 mm ± 1.28 mm; and 2.19 mm ± 1.11 mm, respectively) but the difference between groups was not statistically different (*p*= 0.083; *p*= 0.063; and *p*=0.067, respectively; Fig. 3a).
- PD-reduction. At 12, 18, and 24 months, the reduction was higher in the EMD group (4.25 mm ± 1.06 mm; 4.37 mm ± 1.20 mm; and 4.5 mm ± 0.97 mm, respectively) than in the HA group (3.12 mm ± 1.02 mm; 3.19 mm ± 0.65 mm; and 3.31 mm ± 0.70 mm, respectively). These differences were statistically significant (*p*= 0.005; *p*= 0.003; and *p*= 0.001, respectively; Fig. 3b).
- REC-increase. At 12, 18, and 24 months, the increase in the HA group (0.68 mm ± 0.70 mm; 1.0 mm ± 0.97 mm; and 1.12 mm ± 1.02 mm, respectively) and in the EMD group (1.12 mm ± 0.5 mm; 1.31 mm ± 0.60 mm; and 1.56 mm ± 0.73 mm, respectively) were comparable. These differences were not statistically significant (*p*= 0.056; *p*= 0.247; and *p*= 0.141, respectively; Fig. 3c).
- BOP change. At 12, 18, and 24 months, the BOP change was similar in the HA group (0.06 ± 0.68; 0 ± 0.63; and 0 ± 0.36, respectively) and in the EMD group (0.06 ± 0.68; 0 ± 0.36; and 0.06 ± 0.57, respectively). The minimal observed differences were not statistically significant (*p*= 0.598; *p*= 1; and *p*= 0.695, respectively).

### Frequency distribution

**Fig. 4:**
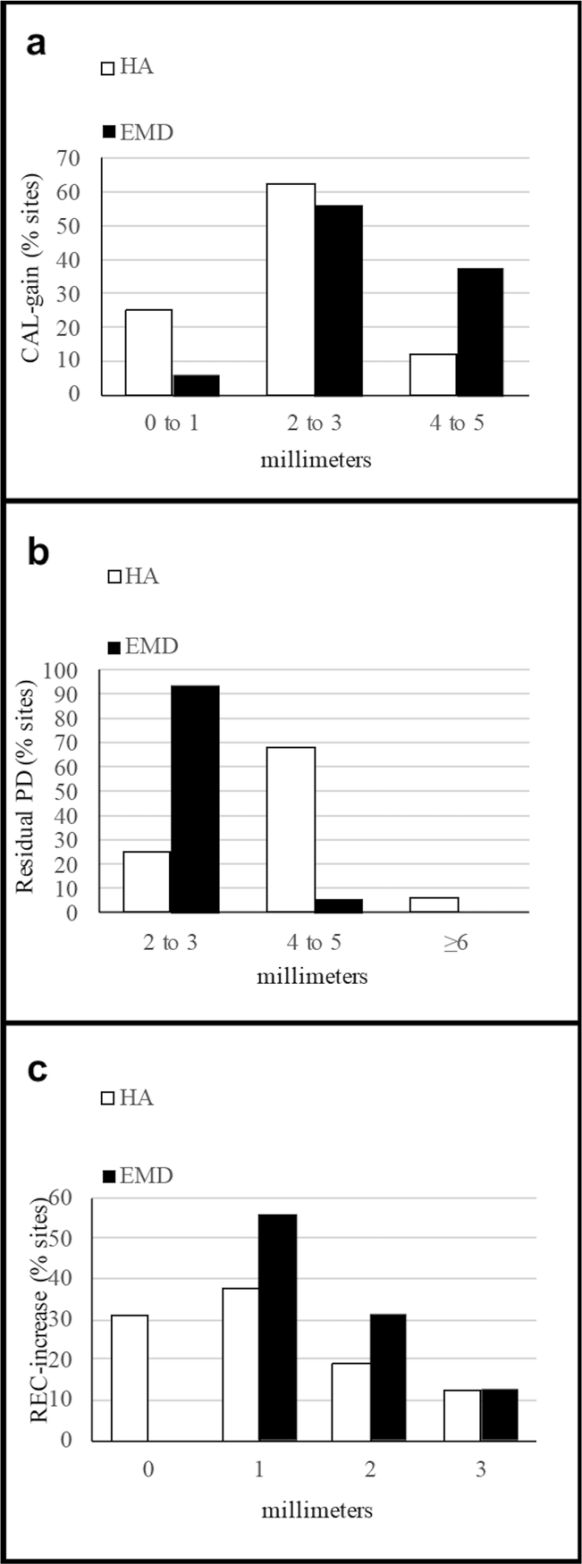
Frequency distribution of clinical parameters changes (expressed as % of sites) at 24 months: **a** CAL-gain, **b** residual PD, **c** REC-increase

Frequency distribution of CAL, PD, and REC changes for both groups at 24 months are shown in Fig. 4.

- CAL. CAL-gain ≤ 3 mm was observed in almost all the HA-treated sites (87.5%, 14 sites) and in 62.5% (10 sites) of the EMD group. Two HA-treated sites (12.5%) gained 4 mm or more while in the EMD group, this value was observed in 6 sites (37.5%) (Fig. 4a).
- PD. Almost the total EMD-treated sites (93.75%, 15 sites) showed residual PD of 2 to 3 mm while in the HA group, this was observed in 25% (4) of the treated sites. A residual PD of 4 to 5 mm was observed in 68.75% (11) HA-treated sites and in only one site in the EMD group. Residual PD ≥ 6 mm was found in 1 HA-treated site and was not observed in the EMD group (Fig. 4b).
- REC. In the HA group, the majority of the sites (68.75%, 11 sites) presented a small increase in gingival recession (≤ 1 mm), while almost half of the EMD-treated sites (43.75%, 7 sites) showed REC-increase ≥ 2 mm (Fig. 4c).

### Treatment-time interactions

The time effects within each treatment group were determined for CAL, PD, and REC. No statistically significant differences were observed between values at 12, 18, and 24 months in the test and control group for CAL (*p*= 0.812 and *p*= 0.893, respectively), PD (*p*= 0.896 and *p*= 0.637, respectively), and REC (*p*= 0.148 and *p*= 0.193, respectively). Furthermore, in both groups and for each of the parameters, no interaction with time was found for any of the comparisons (12 vs 18 months, 12 vs 24 months, and 18 vs 24 months).

## Discussion

EMD has been extensively used in regenerative periodontal surgery and successful short- and long-term clinical results have been reported [4, 5, 26–29, 35]. Based on the outcomes of numerous studies evaluating the use of EMD alone or combined with different biomaterials and surgical approaches, it may be suggested that the use of SFA can be considered a predictable approach for treating intrabony defects with an intrabony component ≥ 3 mm [6, 34]. Interestingly, beneficial effects of EMD on early wound healing have been also reported, although these differences were not different to those reported following the use of barrier membranes [35].

However, despite its widespread use and numerous advantages, it is important to consider a possible limitation of EMD namely the effect of the blood contamination that may negatively influence the adsorption of EMD onto the root surfaces [36, 37]. Therefore, the search for another biomaterial which may overcome this limitation is of clinical interest.

The present randomized trial describes the clinical results at 24 months after the treatment of intrabony defects with HA (test group) and compares the results with EMD (control group). The results have shown favorable clinical outcomes for both treatments in terms of CAL-gain and PD-reduction. However, the test treatment yielded slightly lower REC values while the PD-reduction was statistically significantly higher for the control group. The latter was more evident in the frequency distribution analysis, evaluating the residual PD at 24 months, in which 93.75% of the EMD-treated sites showed residual PD of 2 to 3 mm and 68.75% of the HA-treated sites showed a residual PD of 4 to 5 mm. Noteworthy, no statistically significant differences in terms of the primary outcome measure (CAL-gain) were observed between the two treatments.

HA is a hygroscopic glycosaminoglycan with the ability to modulate wound healing by attracting growth factors and subsequently influencing tissue regeneration [38]. Therefore, the direct contact with the blood does not impair its efficacy. In periodontics, HA has been used for the treatment of gingivitis [39] and periodontitis [40, 41] and more recently also for the treatment of gingival recessions [42–44].

Regarding treatment of intrabony defects, to the best of our knowledge, no studies have been yet published evaluating the outcomes obtained with HA as compared to those following the use of EMD, although a recent systematic review and a meta-analysis [25] revealed better clinical outcomes when HA was used in conjunction with open flap debridement (OFD) as compared to OFD alone.

Briguglio et al. [45], in a randomized clinical study, examined the use of HA to treat intrabony periodontal defects over a period of 24 months in comparison with OFD and concluded that HA shows an additional benefit in terms of CAL-gain and PD-reduction with respect to OFD alone. The mean CAL-gain and PD-reduction reported were 1.9 ± 1.8 mm and 1.6 ± 1.2 mm, respectively. In the present study, the findings regarding the CAL-gain are consistent with the above-mentioned results, whereas the PD-reduction values were higher. However, when interpreting the results, it is important to point out that, in the present study, only defects with an intrabony component of ≥ 3 mm were selected, whereas in the aforementioned trial, defects with a intrabony interproximal component ≥ 5 mm were used. It is well documented that the postoperative CAL-gain and PD-reduction after periodontal treatment or surgical regenerative therapy depends of the baseline characteristics (higher baseline PD, deeper defect, and higher PD-reduction and CAL-gain) [46]. Therefore, considering that the baseline PD and CAL values were similar between our trial and the above-mentioned study, we could expect to obtain lower CAL-gain and PD-reduction values since the treated defects had a shallower intrabony component. However, interestingly opposite outcomes were observed.

A retrospective case series study [47] revealed a mean CAL-gain of 3.8 mm after 1-year which is in line with the present findings and of those obtained with different other regenerative materials [5, 28]. Moreover, in both the above-mentioned studies [45, 47], the REC value was not evaluated, although, from a patient’s perspective, it is one of the major undesirable consequences following periodontal surgical procedures. Therefore, REC was evaluated in the present study and an increase occurred following both treatments. Twenty-four months after surgery, mean REC values were 1.19 ± 0.75 mm for the test and 1.69 ± 0.70 mm for the control group, respectively. This difference was not statistically significant but the *p* value was close to statistical significance (0.052) and it may be of clinical relevance since it may explain, at least in part, the differences observed in the main outcome parameters between the two treatments: the higher PD-reduction (control group) although with no differences in CAL-gain could mean that the highest reduction in the pocket obtained with EDM is due to a greater contraction of the tissues in the healing process, i.e., with increased recession. This was more evident in the analysis of frequency distribution in which 5 out of 16 HA-treated sites not present REC-increase at 24 months and 6 presented REC-increase of 1 mm. Instead, in the EMD group, 9 of 16 sites presented REC of 1 mm.

The effect of HA in reducing gingival bleeding has been previously demonstrated after topical application in patients with gingivitis [48]. However, another study evaluating the effect of HA on the treatment of periodontitis has failed to show any differences in terms of PD and BOP changes after application of HA as compared to the control treatment (i.e., scaling and root planing alone) [49].

In the present study, the primary outcome variable was the difference in CAL-gain between the groups. The reason to select CAL-change as primary outcome variable is based on the fact that this parameter is universally accepted and recommended for studies dealing with periodontal regeneration, in order to perform the power calculation and enable appropriate comparisons between the studies [5, 29]. In the EMD group, the mean CAL-gain value after 24 months was 2.94 ± 1.12 mm and this was comparable with the value reported in previous studies (3.2 mm) [5, 26–28] while in the group treated with HA, the corresponding value was 2.19 ± 1.11 mm. Moreover, due to the finding that at 24 months, no statistically significant difference in terms of CAL-gain was found between the two treatments, and both treatments led to statistically significant CAL-gains compared to baseline, improving over time at each of the evaluation periods, it appears to suggest that HA may be considered a potential option for the surgical treatment of intrabony defects. Nevertheless, the difference in PD-reduction must be also carefully analyzed since this is the only parameter in the present study that presented statistically significant differences between baseline and 24 months between the two treatments (i.e., 3.31 ± 0.7 mm for the test versus 4.5 ± 0.97 mm for control group, respectively). No time effects within each treatment group were detected for CAL, PD, and REC. These results showed that the effects of both treatments were well maintained and neither diminished nor improved during the 24 months. However, previous studies comparing OFD, EMD, and guided tissue regeneration (GTR) have reported that the differences in PD-reduction observed 1 year post-surgical tend to disappear on a long-term basis [28].

Two possible limitations of the present study may be also discussed: (1) the absence of a control group treated with OFD alone, although based on the literature, the use of OFD alone does not seem to be necessary, since several studies have reported the superiority of EMD [5, 26–28] and also of HA [24, 25] when comparing their use in conjunction with OFD versus OFD alone. Thus, based on the available evidence from the literature and keeping in mind the ethical aspect to provide the best treatment option for the patient, the use of OFD alone does not seem to be any longer mandatory as a treatment option for deep intrabony defects [5, 29]; (2) the absence of radiographic bone fill as an outcome parameter: however, the identification and quantification of new bone formation within the treated area remains a challenge. Conventional radiography present limitations regarding bone fill evaluation after periodontal therapy. The image quality, angular distortion, or use of non-standardized imagines can be the main reasons of errors in the assessment of bone gain [50, 51].

In the present study, no radiographic analysis was performed due to the fact that the radiographs were not taken in a standardized way, which would have made an accurate analysis extremely difficult and imprecise, thus introducing a high risk of bias in the study [50, 51]. Furthermore, it is has been repeatedly demonstrated that intraoral radiographs are of limited value to prove periodontal regeneration or formation of a new connective tissue attachment (i.e., formation of cementum and inserting periodontal ligament fibers) since this can be only proven histologically [4, 6]. Data from histological studies have provided evidence for the effect of both EMD and HA to promote formation of cementum and periodontal ligament; however, this may not necessarily be accompanied by bone formation [4, 6, 23, 27, 28]. For these reasons, in the great majority of studies evaluating the outcomes of regenerative periodontal therapy in intrabony defects, no radiographic evaluation was performed [4, 6, 23, 27, 28]. Therefore, in the present study, intraoral radiographs have been only used to illustrate the outcomes of the clinical measurements (i.e., gain of CAL- and PD-reduction) pointing to the potential clinical relevance of the provided treatments.

In summary, the findings of the available in vitro and histological studies [20, 21, 23] together with the obtained clinical improvements reported in the present study appear to suggest that the application of HA in conjunction with OFD facilitates periodontal wound healing in intrabony periodontal defects.

## Conclusion

Within their limits, the present findings indicate that after 24 months, both treatments resulted in statistically significant clinical improvements compared with baseline. Although EMD resulted in statistically significantly higher PD-reduction values compared with HA, the clinical relevance of this difference remains unclear. Therefore, it appears that HA may represent a valuable alternative for treating intrabony periodontal defects in conjunction with a surgical approach. Further clinical studies with a higher number of patients and defects are necessary to confirm the present clinical findings and histologic studies are warranted to evaluate the type of healing obtained following the use of HA when used in conjunction with reconstructive periodontal surgery.
